# Preliminary Outcomes of Arthroscopic Biologic Tuberoplasty in the Treatment of Massive Irreparable Rotator Cuff Tears

**DOI:** 10.7759/cureus.34402

**Published:** 2023-01-30

**Authors:** Raffy Mirzayan

**Affiliations:** 1 Orthopaedic Surgery, Kaiser Permanente Southern California, Baldwin Park, USA

**Keywords:** interpositional tuberoplasty, dermal tuberoplasty, mri in shoulder, superior capsule reconstruction, massive rotator cuff tear, acellular dermal allograft, biologic tuberoplasty

## Abstract

Introduction: Several treatment options exist for the treatment of massive, irreparable rotator cuff tears. A novel technique has been described whereby an acellular dermal allograft is secured to the greater tuberosity which acts as an interpositional tissue preventing bone-to-bone contact between the greater tuberosity and acromion. The preliminary results of this arthroscopic procedure are being presented.

Methods: Patients who underwent a biologic tuberoplasty procedure between 2015 and 2022, by a single surgeon, were included in this study. Pre- and postoperative American Shoulder and Elbow Surgeons (ASES), Single Assessment Numeric Evaluation (SANE), and visual analogue pain scores (VAS) were prospectively recorded and retrospectively reviewed. Postoperative MRI was obtained in some cases. Paired t-test was used to calculate significance set at <0.05.

Results: Ten patients met the inclusion criteria. The mean age was 70.4+4.7 years (range 65-78). There were five males. The mean length of follow-up was 21+27 months (range six to 95 months). There was significant improvement in ASES (24.3+4 to 91.5+10.3, P<0.00001), SANE (22.5+10.3 to 88+11.6, P<0.00001), and VAS (8.8+0.6 to 1.1+2.5, P<0.00001). MRI was obtained in seven patients at a mean of 5.3+2.9 months and showed a healed graft to the tuberosity in all cases.

Conclusion: Biologic tuberoplasty is an effective procedure in improving pain and functional outcomes in patients with massive, irreparable rotator cuff tears.

## Introduction

Several treatment options exist for patients with massive, irreparable rotator cuff tears (MIRCT) including debridement, partial repair, tendon transfer, superior capsule reconstruction (SCR), balloon arthroplasty and reverse shoulder arthroplasty [1-4]. Mihata et al. introduced and popularized SCR for the treatment of MIRCT using tensor fascia lata [3]. Due to concern over donor site morbidity, acellular dermal allograft has become popular in North America [5]. Although the overall outcomes of SCR have been positive, graft tears have been reported [6]. Mirzayan et al. [7] classified graft tear patterns following SCR with dermal allograft into three types: 1. Intact graft, 2. Graft tear from glenoid or mid-substance with a healed graft to the tuberosity (tuberosity covered), and 3. Graft tear from tuberosity or dissolved graft (tuberosity bare). Mirzayan et al. found the clinical outcomes were correlated with graft tear location; patients with an intact graft or a graft tear leaving the tuberosity covered have equivalent improvements from pre-operative state, whereas graft tears leaving the tuberosity uncovered did not show functional or clinical improvement [6,8]. The authors coined the term “biologic tuberoplasty” to describe the effect of the remaining dermal allograft on the tuberosity was acting as an interpositional tissue to prevent bone-to-bone contact between the greater tuberosity and acromion. This led the author to perform biologic tuberoplasty in lieu of SCR for patients with MIRCT, as it was faster, less technically demanding, less costly, and led to much faster recovery [9]. Since then, several authors have described a variation of this technique [10-12]. We present the preliminary clinical and functional outcomes of a series of patients who have undergone biologic tuberoplasty. 

## Materials and methods

After obtaining Kaiser Permanente Southern California Institutional Review Board approval number 13368, patients who had undergone an arthroscopic biologic tuberoplasty using a dermal allograft between 2015 and 2022 were identified. Inclusion criteria included MIRCT, intact or reparable subscapularis, Hamada 1 or 2, no evidence of chondromalacia of the glenohumeral joint. Exclusion criteria included torn or irreparable subscapularis, Hamada 3 or higher, and arthritis of glenohumeral joint. Pre- and postoperative American Shoulder and Elbow Surgeons (ASES), Single Assessment Numeric Evaluation (SANE), and visual analogue pain scores (VAS) were prospectively recorded and retrospectively reviewed. Postoperative MRI was obtained in patients who were willing to undergo an MRI. Clinically significant measures including the minimal clinically important difference (MCID), substantial clinical benefit (SCB), and patient acceptable symptom state (PASS) were calculated to reflect patient benefit and satisfaction after surgery. The MCID establishes the change in outcome score that results in the smallest, appreciable clinical improvement after surgery, the SCB demonstrates further improvement that a patient finds to be considerable, and the PASS represents the level of postoperative outcome score required [13]. The MCID, SCB, and PASS used for ASES were 11.1, 17.5, and 86.7, respectively, and for SANE were 16.9, 29.8, and 82.5, respectively [13].

Surgical technique

**Figure 1:**
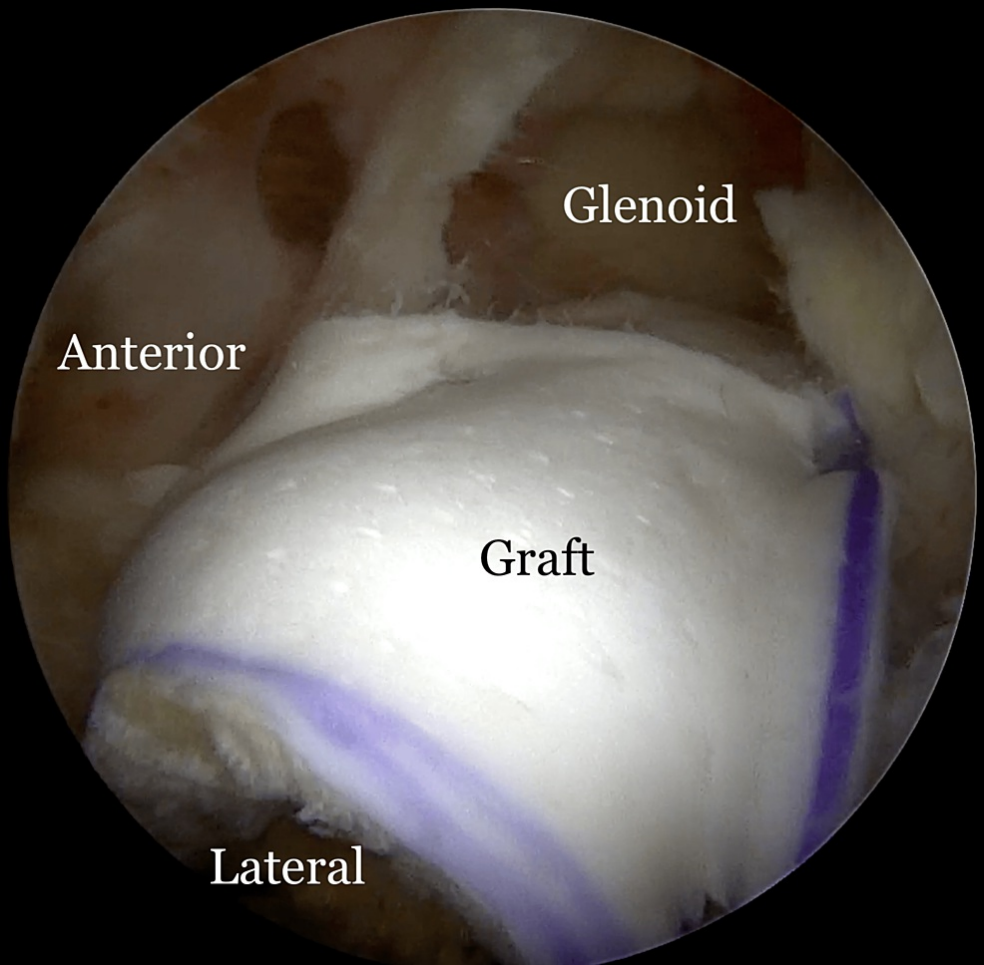
Biologic tuberoplasty with a dermal allograft Arthroscopic view of a left shoulder following biologic tuberoplasty with a dermal allograft (ArthroFlex 301; LifeNet Health, Virginia Beach, VA, USA).

The variations in technique have been previously described [9-12]. Briefly, the greater tuberosity is prepared by removing soft tissue with a radiofrequency wand or a burr in reverse mode. If large osteophytes exist from the tuberosity, they are excised with a bur. However, care is taken not to aggressively decorticate the tuberosity. The length and width of the tuberosity footprint are measured. On the back table, a 3mm acellular dermal allograft (ArthroFlex; LifeNet Health, Virginia Beach, VA, USA) is cut to size approximately 15% smaller than the measured dimensions of the greater tuberosity as the graft tends to stretch. Typically, the graft dimensions are 25mm long (anterior to posterior) and 15 to 20mm wide (medial to lateral). Once the graft has been oriented, two FiberLink sutures (Arthrex, Naples, FL, USA) are “luggage tagged” in the anterolateral and posterolateral corners for future fixation in the lateral row. Two knotless anchors (FiberTak; Arthrex) are placed medially off the articular margin of the humeral head. One is placed just posterior to the biceps (anteromedial anchor) and the other as far posterior as possible to cover the entire anteroposterior length of the tuberosity (posteromedial anchor). The knotless sutures are then retrieved through a lateral PassPort cannula (Arthrex) with a divider to keep the anterior and posterior sutures apart. The repair stitch from the anterior anchor is then passed through the graft in the anteromedial aspect of the graft in a mattress fashion. The repair stitch is then passed through the loop stitch from the same anchor and the pulling stitch is pulled to bring the repair stitch into the anteromedial anchor, locking it in a finger trap fashion. The same step is repeated with the posterior sutures through the posteromedial aspect of the graft. When the pull stitches from the two medial anchors are pulled, the graft is reduced into the subacromial space through the cannula and secured to the medial row. The two luggage tag sutures are then anchored laterally using either PushLock or SwiveLock anchors (Arthrex) (Figure 1, Video 1).

**Video 1:** Biologic tuberoplasty with a dermal allograft Arthroscopic view of a left shoulder with a massive retracted irreparable rotator cuff tear. The greater tuberosity is covered with a dermal allograft in order to prevent bone-to-bone contact between the tuberosity and the acromion.

Postoperative protocol

The patient is kept in a sling for three weeks. Beginning on week four, the sling is removed and active and active assist range of motion exercises are initiated without any limitations in range of motion. At six weeks (and typically sooner), full range of motion is achieved, and strengthening is initiated and advanced as tolerated without any restrictions.

Statistical analysis

A paired student t-test was performed to compare the matched pair means of the pre- and postoperative ASES, SANE, and VAS scores. Statistical significance was set at a p value < 0.05. 

## Results

Ten patients underwent biologic tuberoplasty by a single surgeon. The mean age was 70.4+4.7 years (range 65-78). There were five males. The mean length of follow-up was 21+27 months (range: six to 95 months). Nine patients were Hamada 1, and one patient was Hamada 2. Eight patients had a concomitant subpectoral biceps and two patients also had a distal clavicle resection. All patients had an intact subscapularis. The mean graft dimensions were 26.5+3.4mm in length (anterior to posterior direction) and 18+2.6mm in width (medial to lateral direction). 

There was significant improvement in ASES (24.3+4 to 91.5+10.3, P<0.00001), SANE (22.5+10.3 to 88+11.6, P<0.00001), and VAS (8.8+0.6 to 1.1+2.5, P<0.00001) (Table 1). MRI was obtained in seven patients at a mean of 5.3+2.9 months and showed a healed graft to the tuberosity in all cases. Using clinical thresholds for ASES, 100% achieved MCID, 100% achieved SCB, and 90% achieved PASS. With thresholds for SANE, 100% achieved MCID, 100% achieved SCB, and 80% achieved PASS. There were no complications, including infection or inflammatory response.

**Table 1:** Patient demographic and outcome data ASES- American Shoulder and Elbow Surgeons, SANE- Single Assessment Numeric Evaluation, VAS- Visual Analog Scale, F- Female, M- Male, S- Satisfied, U- Unsatisfied, SD- standard Deviation

Patient	Age	Sex	Length of Graft	Width of Graft	Pre ASES	Pre SANE	pre VAS	post-op ASES	Post SANE	Post-op VAS	Satisfied?	Follow Up (months)
1	71	F	25	15	26	20	9	97	95	0	S	95.1
2	69	M	25	20	25	30	8	100	90	0	S	23.5
3	78	M	30	20	28	40	8	100	100	0	S	20.8
4	69	M	30	20	22	20	9	100	100	0	S	21.7
5	66	F	25	15	17	0	10	93	90	0	S	12.3
6	79	F	30	20	25	20	9	97	90	0	S	10.5
7	71	F	30	20	32	20	8	85	80	2	S	8.5
8	68	F	20	20	23	30	9	68	60	8	U	6
9	65	M	25	15	22	20	9	83	85	1	S	6
10	68	M	25	15	23	25	9	92	90	0	S	6
Mean	70.4		26.5	18	24.3	22.5	8.8	91.5	88.0	1.1		21.0
SD	4.7		3.4	2.6	4.0	10.3	0.6	10.3	11.6	2.5		26.9

## Discussion

Massive, irreparable rotator cuff tears (MIRCT) are a challenging problem to treat with several options being available [1-4]. One must ask the question “where does the pain come from in shoulders with MIRCTs?” While several sources have been implicated, one possibility is from bone-to-bone contact between the acromion and greater tuberosity, leading to pain and acetabularization of the acromion over time [14]. Burkhart et al. [15] described the suspension bridge model in patients with massive, irreparable rotator cuff tears. Those shoulders that had a captured fulcrum developed an acromiohumeral fulcrum, moving the center of rotation superiorly with deltoid contraction developing an acromiohumeral articulation leading to bone-to-bone contact. Thus, preventing bone-to-bone contact between the tuberosity and acromion will theoretically prevent pain.

Superior capsule reconstruction (SCR) using autologous tensor fascia lata was introduced by Mihata et al. [3], but the use of dermal allograft gained popularity in the United States [5]. While clinical results of SCR using dermal allograft have been promising, the procedure is technically demanding, time-consuming, and costly. Because of these factors, the number of SCRs being performed in the United States is declining [16]. Moreover, graft tears have been reported [17,18]. Mirzayan et al. [7] have classified graft tear patterns into three categories: 1. Intact graft 2. Graft tear (from glenoid or mid-substance) leaving the tuberosity covered with dermal allograft, and 3. Graft tear (from tuberosity or dissolved graft) leaving the tuberosity bare. Mirzayan et al. [6,8] reported that those patients with a graft tear leaving the tuberosity covered had significant improvements from the preoperative state, and equivalent outcomes compared to those with an intact graft. Those with a graft tear leaving the tuberosity bare did not have significant improvements. Mirzayan et al. [6] coined the term “biologic tuberoplasty” effect because the dermal allograft was preventing bone-to-bone contact between the greater tuberosity and the acromion, thus eliminating pain [19]. This led Mirzayan et al. [9,20] to perform the biologic tuberoplasty procedure, instead of SCR, for patients with MIRCT by securing dermal allograft to the greater tuberosity with anchors.

Isolated tuberoplasty procedure, as initially described by Fenlin et al. [21], was mini-open and consisted of rounding of the greater tuberosity to create a congruent acromiohumeral articulation, and it has been shown to have acceptable improvements in pain and function at a mean follow-up of 27 months. However, continuation of superior migration of the humeral head has been noted, and other long-term studies have shown borderline satisfactory outcomes [22-25]. Lee et al. [23] reported on 32 patients who underwent arthroscopic tuberoplasty and were followed for a mean of 40 months. The University of California, Los Angeles (UCLA) score improved from a preoperative mean of 15.4 points to 27.1 points, which was significant. However, a UCLA score of >27 (out of 35) is considered satisfactory [26], and this cohort barely reached that criteria. Park et al. [22], from the same institution as the previous report [23], reported on only 16 patients (16 patients were lost to follow-up) who underwent arthroscopic tuberoplasty with a mean of 98 months follow-up. Although there was significant improvement, with the mean UCLA score improving from 10.3 to 27.2, the final score barely met as a satisfactory outcome as described above. In addition, since half their cohort was lost to follow-up, these results could be biased towards those who continued to do well at longer follow-up. It is challenging to compare the results of our study where a dermal allograft was applied to the greater tuberosity with these studies where only a tuberoplasty was performed, as the same patient-reported outcome tools were not used. However, our results had near excellent ASES and SANE scores, with minimal to no pain at follow-up. It appears that the addition of a dermal allograft to the tuberosity seems to have an added advantage to tuberoplasty alone.

Cventanovic et al. [13] established clinically significant outcomes for rotator cuff repairs. The MCID establishes the change in outcome score that results in the smallest, appreciable clinical improvement after surgery, the SCB, is considerable improvement by patient, and the PASS reflects patient satisfaction represented by the level of postoperative outcome score. These metrics represent tiers of health states, where achieving MCID represents minimal improvement from preoperative health, achieving SCB represents substantial improvement from preoperative health, and PASS represents patient satisfactory outcomes. All our patients met the MCID and SCB for both ASES and SANE scores, with only one patient having an unsatisfactory PASS score for ASES and two patients for SANE.

This study has some limitations. There are a limited number of patients, hence this is only a preliminary report. The mean follow-up is 21 months, with a minimum of six months. Long-term follow-up is needed. However, one patient with eight-year follow-up had maintained her clinical outcomes with great satisfaction.

## Conclusions

We report the preliminary outcomes of arthroscopic biologic tuberoplasty. This is a novel procedure where an acellular dermal allograft is secured to the tuberosity, covering the entire footprint. This biologic tissue is permanent and prevents bone-to-bone contact between the tuberosity and acromion. Our results show that this procedure, in a limited series of patients, has been proven to be effective in relieving pain and improving functional outcomes in patients with massive irreparable rotator cuff tears. This technique is faster, less technically demanding and much quicker rehabilitation and recovery compared to superior capsule reconstruction. Prospective studies with a higher number of patients, or prospective randomized trials comparing biologic tuberoplasty versus debridement, should be performed to validate the findings of this preliminary study. 
